# Protective Effect of *Apocynum venetum* L. Leaves Extract Against Diabetic Cardiomyopathy: Inhibition of Oxidative Stress and Ferroptosis via Modulation of the Xc^−^/GSH/GPX4 Axis

**DOI:** 10.3390/cimb48040375

**Published:** 2026-04-03

**Authors:** Subinuer Abuduaini, Guohua Shi, Li Chen, Subinuer Erreken, Mei Long, Xiaoqian Tang, Jinsen Kang

**Affiliations:** 1 College of Pharmacy, Xinjiang Medical University, Urumqi 830011, China; subinr15@stu.xjmu.edu.cn (S.A.); sgh990331@stu.xjmu.edu.cn (G.S.); 002661su@stu.xjmu.edu.cn (S.E.); longmei0217@stu.xjmu.edu.cn (M.L.); txq20001106@stu.xjmu.edu.cn (X.T.); 2 Xinjiang Technical Institute of Physics and Chemistry, Chinese Academy of Sciences, Urumqi 830011, China; chenli@ms.xjb.ac.cn

**Keywords:** *Apocynum venetum* L. leaves, diabetic cardiomyopathy, ferroptosis, Xc^−^/GSH/GPX4 axis, oxidative stress, mitochondrial damage

## Abstract

Background: Diabetic cardiomyopathy (DCM), a common cardiovascular complication associated with diabetes mellitus, has the potential to progress to heart failure. *Apocynum venetum* L. leaves extract (AVLE) possesses known cardioprotective activity, but its effect on DCM remains unclear. This study explored the protective effects of AVLE against myocardial injury in type 2 diabetes and the underlying mechanisms. Methods: DCM was established in vivo using db/db mice and in vitro using high-glucose, high-fat (HGHF)-stimulated H9c2 cardiomyocytes. We evaluated metabolic profiles, cardiac function, histopathology, oxidative stress, inflammation, and ferroptosis. Results: In vivo, following 12 weeks of AVLE treatment, cardiac function and structural integrity were significantly improved, serum cardiac injury markers and dyslipidemia were reduced, and pathological myocardial remodeling was attenuated in db/db mice; in vitro, AVLE enhanced cell viability and attenuated cellular damage under HGHF conditions. Mechanistically, AVLE alleviated oxidative stress and inflammation, restored mitochondrial function, and inhibited ferroptosis by regulating key pathway proteins; it upregulated GPX4 and SLC7A11, while downregulating TfR1 and ACSL4. Conclusions: AVLE exerts cardioprotective effects against diabetic cardiomyopathy by reducing oxidative stress and inflammation, mitigating lipid peroxidation and mitochondrial damage, ultimately inhibiting ferroptosis through regulation of the Xc^−^/GSH/GPX4 axis.

## 1. Introduction

In recent years, with improvements in people’s material living standards and lifestyle changes, metabolic diseases like type 2 diabetes mellitus (T2DM) have been experiencing a global surge. In 2024, the prevalence of diabetes among adults aged 20–79 years reached an alarming figure of roughly 589 million individuals worldwide. This statistic, highlighted in the 11th edition of the IDF Diabetes Atlas, underscores a significant global health concern. Notably, more than 90% of these diabetes cases are classified as type 2 [1,2], indicating that this particular form of diabetes represents the majority of instances diagnosed across the population. The findings in the IDF Diabetes Atlas emphasize the urgent need for effective prevention and management strategies. Being overweight or obese and having abnormal blood lipid levels are all high-risk factors for diabetes. Elevated blood glucose and lipid levels have become a global health concern, contributing to damage in macrovascular, microvascular, cardiac, cerebral, renal, and other organ systems. Diabetic cardiomyopathy (DCM), first described by Rubler et al. in 1972 [3], is defined as a specific form of myocardial disease characterized by structural and functional cardiac changes directly caused by diabetes, while excluding the presence of hypertension, valvular heart disease, or other known cardiac disorders. As DCM progresses, cardiomyocyte hypertrophy and myocardial fibrosis contribute to ventricular wall thickening and reduced ventricular compliance. This gradually leads to impaired cardiac systolic function, typically evidenced by a reduced ejection fraction, and may eventually result in heart failure or even death [4]. Glucose and lipid metabolic disorders constitute a significant contributor to the progression of DCM. Chronic exposure of cardiomyocytes to high glucose not only exerts direct glucotoxicity and lipotoxicity effects but also indirectly impairs cardiac structure and function by triggering inflammation, mitochondrial dysfunction, oxidative stress, and myocardial fibrosis [5,6,7]. Research has indicated that the application of certain classic antidiabetic drugs has not markedly reduced mortality from cardiovascular complications in diabetic patients and may even elevate heart failure risk [8]. Therefore, relying solely on lowering blood glucose may not fully prevent myocardial damage caused by hyperglycemia. Additionally, because of its intricate pathogenesis involving multiple factors, the development of therapeutic strategies targeting its core pathological mechanisms remains a significant and unsatisfied clinical need. This emphasizes the need to explore novel prevention and treatment strategies for DCM. Based on multi-target regulation and lower side effects, natural products from traditional Chinese medicine offer a highly promising research pathway. Focused on an unmet clinical need, this research evaluates natural products as a basis for novel therapeutic approaches to control diabetes and mitigate its complications.

Ferroptosis represents a distinct form of programmed cell death that is specifically marked by the excessive accumulation of lipid peroxides due to iron mediation [9]. In contrast to other typical forms of non-programmed cell death, ferroptosis is primarily associated with iron metabolism disorders, lipid peroxidation, and imbalances in the antioxidant system [10]. These unique characteristics underscore the importance of understanding ferroptosis in the context of various diseases and cellular dysfunctions. Emerging findings position ferroptosis as a key pathological mediator in various cardiac diseases. Elevated iron levels, high glucose, and increased fatty acid oxidation drive ROS generation, which induces oxidative stress and subsequently accelerates the progression of diabetes and its associated complications [11,12]. Du et al. [13] confirmed that canagliflozin inhibits ferroptosis in T1DM mice by balancing myocardial iron homeostasis, alleviating oxidative stress, and modulating the Xc^−^/GSH/GPX4 axis, which is the principal pathway protects against ferroptosis, in normal physiological conditions, by mediating the transformation of harmful lipid hydroperoxides into harmless lipid alcohols. Xu et al. [14] established a model of obesity-induced myocardial injury using a high-fat diet and demonstrated that finerenone mitigates cardiac lipotoxicity primarily by inhibiting ferroptosis, a mechanism mediated through its modulation of the Xc^−^/GSH axis. The study by Chen et al. [15] revealed that nicorandil mitigates DCM through the inhibition of ferroptosis via the AMPK–Parkin–ACSL4 signaling pathway. These findings further indicate that therapeutic approaches designed to suppress ferroptosis offer novel options for the prevention and treatment of DCM.

*Apocynum venetum* L., a perennial herbaceous plant of the Apocynaceae family, has been traditionally used in the production of herbal medicines and tea. Since 2002, *Apocynum* tea has been listed as a health food in China [16]. Recent studies on chemical composition and pharmacology indicate that *Apocynum venetum* leaves are rich in flavonoids and flavonoid glycosides, steroids, fatty acids, tannins, and other glycosides. These compounds possess various previously reported pharmacological activities, such as antihypertensive, lipid-lowering, cardioprotective, hepatoprotective, anti-inflammatory, and antioxidant effects [17]. Zhao et al. [18] showed that AVLE inhibits DOX-induced cardiac toxicity by reducing LDH, CK, and BNP levels in mice, while inhibiting apoptosis and myocardial fibrosis. The findings of Xie et al. [19] indicate that AVLE provides protective effects against verapamil-hydrochloride-induced cardiac injury in zebrafish. Xu et al. [20] demonstrated that flavonoid-enriched AVLE decreased serum and hepatic tissue TC, TG, and LDL-C levels in a rat model of hyperlipidemic. It also mitigated vascular endothelial injury induced by hyperlipidemia and ameliorated oxidative stress levels in vivo.

Our research group maintains a long-standing dedication to the development and functional evaluation of bioactive compounds from AVLE. We have previously established a stable extraction and preparation process for the AVLE used in this study. Using UHPLC-MS, we systematically characterized its chemical composition, identifying 62 compounds (including 41 flavonoids, 17 phenolic acids, and other compounds). The content of major components such as isoquercitrin and rutin was clearly determined [21]. This previous work, combined with published data, offers a solid theoretical foundation for this study. Although these validated bioactivities suggest potential protective effects against diabetic myocardial injury, the specific protective mechanisms of AVLE in diabetic cardiomyopathy (DCM) require further investigation.

Therefore, this study employed a strategy using both animal (in vivo) and cellular (in vitro) systems to elucidate the cardioprotective roles of AVLE against diabetic cardiomyopathy in db/db mice and against HGHF-induced damage in H9c2 cardiomyocytes. It elucidates the potential mechanisms, verifies whether AVLE exerts its protective effects by inhibiting the ferroptosis pathway, and thereby provides scientific evidence to support the subsequent drug development and clinical application of *Apocynum venetum* L. leaves extracts.

## 2. Materials and Methods

### 2.1. Drugs and Reagents

*Apocynum venetum* L. leaves extraction was provided by the Xinjiang Technical Institute of Physics and Chemistry (No.: 20240511) [21]. Cell culture reagents, including Dulbecco’s modified Eagle’s medium (DMEM, 11885500BT), fetal bovine serum (FBS, 10099-141C), penicillin/streptomycin (15140-122), and 0.25% Trypsin-EDTA (C25200-056) sourced from Gibco (Waltham, MA, USA). D-(+)-Glucose (purity ≥ 99.5%, G7021) was obtained from Sigma-Aldrich (St. Louis, MO, USA). Palmitic acid (PA, KC004) was purchased from Kunchuang (Xi’an, China). Metformin (purity ≥ 98.0%, D9351) was obtained from Solarbio (Beijing, China). Ferrostatin-1 (HY-100579) was purchased from MedChemExpress (MCE, Shanghai, China).

### 2.2. Preparation of AVLE

*Apocynum venetum* leaves were identified by Dr. Lu Chunfang from Xinjiang Technical Institute of Physics and Chemistry. Preparation of AVLE followed a method detailed in our prior work [21]. In brief, leaves (250 g) were subjected to dual cycles of continuous reflux extraction using 50% (*v*/*v*) ethanol (7500 mL per cycle, 1 h each). The extracts were filtered, concentrated in vacuo, and lyophilized. For purification, the extract (reconstituted at 0.1 g/mL) was loaded onto an HPD300 macroporous resin column. Sequential elution with 1 BV of water and 4 BV of 50% ethanol was performed to remove impurities and collect the target fraction, respectively, which was then concentrated and dried to yield the purified extract. Chemical characterization of AVLE by UHPLC-MS is provided in the Supplementary Materials (Figure S1 and Table S1) [21].

### 2.3. Animals

Six-week-old db/m (22 ± 2 g), db/db mice (32 ± 2 g), male, were acquired from CAVENS (SCXK(Su)2021-0013; Changzhou, China). The study was conducted at the SPF-grade Experimental Animal Center, where mice were maintained in standardized housing (22 ± 2 °C, 55 ± 5% humidity, 12-h light/dark cycle) with ad libitum access to food and water. Six db/m mice served as the control group (CON), and thirty db/db mice were randomly assigned to 5 groups (n = 6): the model group (MOD), the low-dose (AVLE-L, 140 mg/kg/d) group, the moderate-dose (AVLE-M, 280 mg/kg/d) group, the high-dose AVLE (AVLE-H, 560 mg/kg/d) group, and the metformin positive group (MET, 200 mg/kg/d). Daily gastric administration was performed at a fixed time, and body weight and fasting blood glucose (AccuChek^®^ Performa Combo, Roche, Indianapolis, IN, USA) were measured every 2 weeks. All groups received continuous gastric treatment for 12 weeks.

### 2.4. Cell Culture

H9c2(2-1) cells (GDC0606) were sourced from the China Center for Type Culture Collection (CCTCC, Wuhan, China), cultured in DMEM containing 10% FBS and 1% penicillin/streptomycin at 37 °C under a 5% CO_2_ incubator. To induce DCM model, the cells were exposed to DMEM containing 35 mM glucose + 0.1 mM palmitic acid (PA). The subsequent experiments will consist of the following six groups: CON group: 5.5 mM glucose, HGHF group: 35 mM glucose + 0.1 mM PA, AVLE-L group: HGHF + 10 μg/mL AVLE, AVLE-M group: HGHF + 20 μg/mL AVLE, AVLE-H group: HGHF + 40 μg/mL AVLE, and Fer-1 group: HGHF + 2 μM Fer-1. Each group was treated for 24 h.

### 2.5. Echocardiography

After the last treatment, the mice in all groups were anesthetized by isoflurane inhalation (RWD, Shenzhen, China). The mice’s chest hair was then removed with depilatory cream. Each mouse was positioned to ensure even application of the ultrasound coupling agent, and B-mode ultrasound images were acquired using a parasternal long-axis view of the left ventricle. LVAW, LVPW, and LVID were measured. LVFS and LVEF were calculated to assess systolic–diastolic function.

### 2.6. Sample Collection

Serum: After anesthetizing the mice, blood was collected. After settling for 2 h, serum was then collected after centrifugation and stored at −80 °C. For cardiac tissue, left ventricular heart tissues were fixed in 4% paraformaldehyde or 2.5% glutaraldehyde (G1101/G1102, Servicebio, Wuhan, China). Residual tissues were snap-frozen in liquid nitrogen.

### 2.7. Histopathology

The collected cardiac ventricular tissues were placed in 4% paraformaldehyde for over 24 h, followed by paraffin embedding and sectioning at a thickness of 5 μm. Histopathological features and collagen fiber deposition in heart tissues were evaluated through H&E and Masson staining, and WGA staining was used to observe myocardial hypertrophy.

### 2.8. Transmission Electron Microscopy

Samples were processed through fixation, dehydration, and impregnation before embedding in epoxy and acrylic resins, from which 1 mm thick sections were sliced from the embedded samples using an ultramicrotome equipped with glass knives. The ultrastructure of myofilaments and mitochondria was examined under a transmission electron microscope for subsequent morphological analysis (HT7800, Hitachi, Tokyo, Japan).

### 2.9. Cell Viability

The CCK-8 assay kit (E-CK-A362, Elabscience, Wuhan, China) was used to assess cell viability. H9c2 cells were seeded at 5 × 10^4^ cells/mL in a 96-well plate. After 24 h of adhesion, the cells were administered with graded doses of AVLE or PA for 24 h. Subsequently, 100 µL of DMEM containing 10% CCK-8 was applied, After 2 h at 37 °C, absorbance at 450 nm was determined.

### 2.10. Biochemical Analysis

Serum levels of TC, TG, LDL-C, HDL-C, CK-MB, and LDH were determined using a fully automated blood biochemistry analyzer (Chemray800, Rayto, Shenzhen, China). Oxidative stress and lipid peroxidation biomarkers were detected using kits purchased from Solarbio, China: MDA (BC0025), SOD (BC5165), and GSH (BC1175). The LDH assay kit (A020-2-2, Nanjing Jiancheng, Nanjing, China) for detecting LDH in H9c2 cell supernatants. The Fe^2+^ assay kit (E1046) for measuring Fe^2+^ levels was purchased from Prilay (Beijing, China). Procedures followed the manufacturer’s instructions, and corresponding absorbance values were obtained with multimode microplate reader (Tecan, fusion-fx6 edge v.070, Männedorf, Switzerland).

### 2.11. ELISA

TNF-α, IL-6, and IL-1β levels were detected in mouse hearts and cell culture supernatants using kits purchased from FineTest (Wuhan, China). Procedures followed the manufacturer’s instructions, and corresponding absorbance values were obtained with multimode microplate reader.

### 2.12. Proteomics

Heart tissue was used for protein extraction, and protein concentration was determined via BCA assay. A 60 μL sample was reduced with dithiothreitol, alkylated with iodoacetamide, and then digested into peptides with trypsin. After the digestion process, the mixture was vacuum-dried and resuspended for instrumental analysis, followed by analysis using nano-LC-MS/MS. Raw MS data were processed with DIA-NN (1.8.1) software and searched against the UniProt database, with a FDR threshold set at ≤0.01 for peptides and peptide-spectrum matches (PSMs). Differentially expressed candidate proteins were identified via *t*-tests (*p* < 0.05). Bioinformatics analysis of proteins with significant changes was then performed using enrichment studies in the GO and KEGG databases.

### 2.13. Fluorescence Staining Assay

Superoxide production, mitochondrial membrane potential (MMP), and ferrous ion (Fe^2+^) levels were detected using the DCFH-DA (E-BC-K138-F, Elabscience, Wuhan, China), JC-1 (C2006, Beyotime, Shanghai, China), and FerroOrange (F374, Dojindo, Kumamoto, Japan) fluorescent probes, respectively. Following modeling and drug treatment as detailed in the Section 2.4, cells were rinsed with PBS and incubated with fluorescent probes (5 µM DCFH-DA, 10 µM JC-1, or 1 µM FerroOrange) at 37 °C under dark conditions for 30 min, following three rinses with related staining buffer, then visualized under a fluorescence microscope. Finally, to facilitate a precise measurement of the levels of fluorescence corresponding to each group, the intensity was quantified with Image J (v.1.54g, National Institutes of Health, Bethesda, MD, USA).

### 2.14. Western Blot

Total protein extraction from myocardial tissue and H9c2 cells was performed utilizing RIPA lysis buffer (R0020, Solarbio) containing 1% protease inhibitor (M5293, Abmole, Houston, TX, USA); protein concentration was quantified with BCA assay (PC0020, Solarbio, China). Proteins were separated through SDS-PAGE, transferred onto PVDF membranes (Immobilon, IPVH00010, Millipore, Tullagreen, Ireland), and blocked using 5% skim milk (1172GR, BioFrox, Einhausen, Germany) for 2 h. After overnight incubation with primary antibodies at 4 °C and rinses with TBST, the membranes were incubated with secondary antibodies for 1 h. Following a final TBST rinse, protein bands were visualized with an ultra-sensitive ECL chemiluminescent reagent (PK10003, Proteintech, Wuhan, China). Detection of the bands was achieved with a gel imaging system, which provides clear images for analysis. Finally, the blots were quantified using Image J. The antibodies used in this study were anti-FTH1 (1:4000, 11682-1-AP), anti-β-actin (1:10,000, 81115-1-RR), and HRP-goat anti-rabbit secondary antibody (1:8000, RGAR001), which were acquired from Proteintech (Wuhan, China). Anti-GPX4 (1:1000, ET1706-45), anti-SLC7A11 (1:1000, HA721868), anti-TfR1 (1:3000, ET1702-06) and anti-ACSL4 (1:3000, ET7111-43) were acquired from HUABIO (Hangzhou, China).

### 2.15. Statistical Analysis

All statistical analyses were conducted utilizing GraphPad Prism 9.0. Data are expressed as mean ± SEM. Normality was assessed with the Shapiro–Wilk test. For two-group comparisons, two-tailed Student’s *t*-test was applied; for multiple groups, one-way ANOVA with Tukey’s post hoc test was used. A *p*-value of less than 0.05 was considered to indicate statistical significance in the findings.

## 3. Results

### 3.1. Effects of AVLE on Diabetic Myocardial Injury in db/db Mice

**Figure 1:**
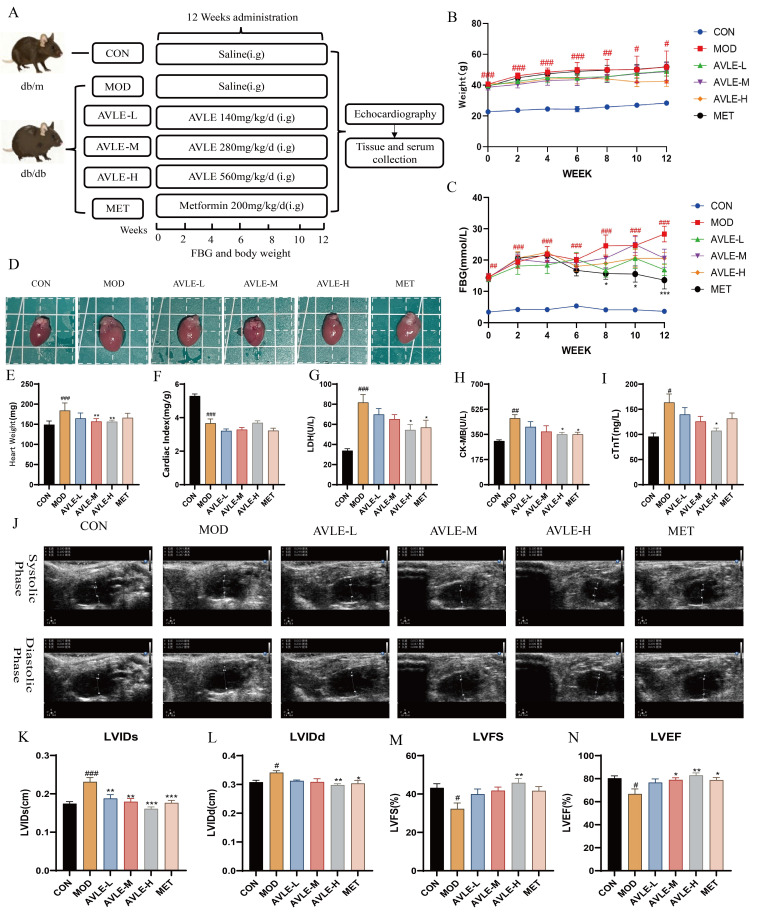
AVLE attenuates myocardial injury in db/db mice. (**A**) Experimental assignment of groups; (**B**) Body weight; (**C**) FBG; (**D**) Gross appearance of the heart; (**E**) Heart weight; (**F**) Cardiac index; Serum LDH (**G**), CK-MB (**H**) and cTnT (**I**) level; (**J**) Echocardiographic images, The measurement indicates length (cm); Quantification of LVIDs (**K**), LVIDd (**L**), LVFS (**M**), and LVEF (**N**). Data are reported as mean ± SEM (n = 6, # *p* < 0.05, ## *p* < 0.01, ### *p* < 0.001 vs. CON; * *p* < 0.05, ** *p* < 0.01, *** *p* < 0.001 vs. MOD).

A DCM model was established using db/db mice, in which the MOD group exhibited significantly elevated FBG and body weight, along with markedly increased cardiac weight and cardiac index. Furthermore, cardiac injury biomarkers LDH, CK-MB, and cTnT were significantly elevated in serum, while LVIDd and LVIDs were markedly elevated, LVEF and LVFS were significantly reduced. These findings suggest myocardial injury and impaired systolic–diastolic function in db/db mice. Following 12 weeks of intervention, MET significantly reduced FBG levels. Heart weights were markedly decreased in the AVLE-M and AVLE-H groups (*p* < 0.05, Figure 1E). Serum LDH and CK-MB levels were markedly decreased in the AVLE-H and MET groups, while cTnT levels were markedly reduced in the AVLE-H group (*p* < 0.05, Figure 1G–I). The AVLE-H group also showed significantly reduced LVIDd and LVIDs, along with significantly increased LVEF and LVFS. The MET group showed significantly reduced LVIDs and LVIDd, and significantly increased LVEF (*p* < 0.05, Figure 1J–N). These results indicate that AVLE alleviates diabetic myocardial injury and restores systolic–diastolic function in db/db mice.

### 3.2. Effects of AVLE on Histopathology, Lipid Metabolism, Oxidative Stress, and Inflammation in db/db Mice

**Figure 2:**
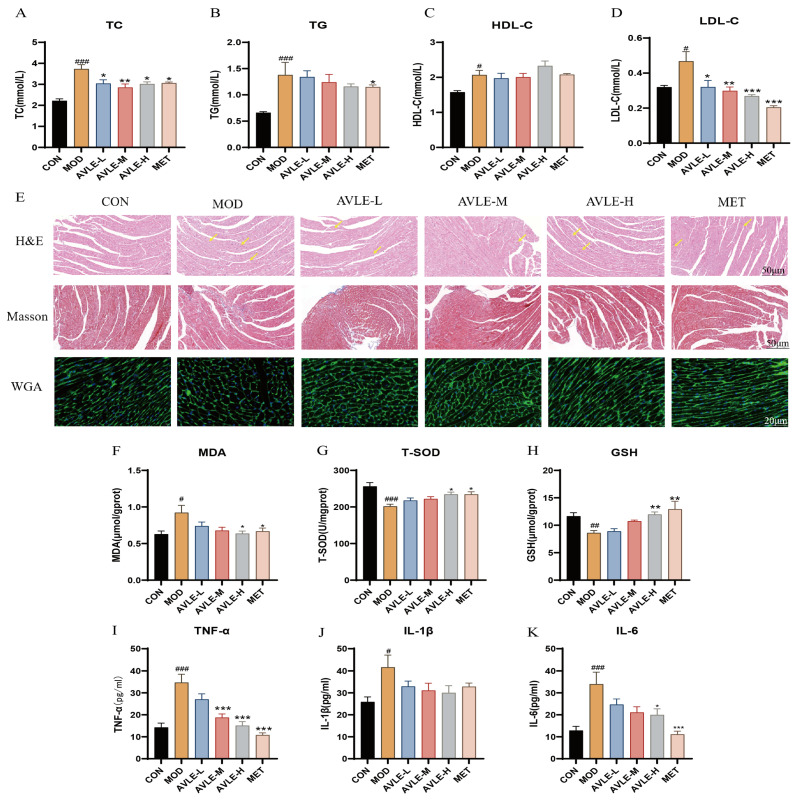
Effects of AVLE on Lipid Metabolism, Oxidative Stress, Inflammation, and Cardiac Remodeling in db/db Mice. Contents of (**A**) TC; (**B**) TG; (**C**) HDL-C and (**D**) LDL-C in serum; (**E**) Pathological changes in myocardial tissues (scale bar: 50 μm/20 μm); Contents of (**F**) MDA; (**G**) T-SOD; (**H**) GSH in myocardial tissues; Contents of (**I**) TNF-α; (**J**) IL-1β; (**K**) IL-6 in myocardial tissues. Data are reported as mean ± SEM (n = 6, # *p* < 0.05, ## *p* < 0.01, ### *p* < 0.001 vs. CON; * *p* < 0.05, ** *p* < 0.01, *** *p* < 0.001 vs. MOD).

Lipid function test results indicate that serum TG, TC, and LDL-C levels showed a significant increase in the MOD group. Following 12 weeks of intervention, TC and LDL-C levels decreased significantly by 19% and 42.5% in the AVLE-H group, respectively (*p* < 0.05, Figure 2A,D), while TG levels decreased by 15.9% and HDL-C levels increased by 12.1% (Figure 2B,C). These findings suggest that AVLE modulates lipid metabolic processes in db/db mice.

Histopathological staining showed that in the MOD group, cardiomyocytes exhibited disorganized, numerous vacuolar degeneration (yellow arrows), with marked collagen accumulation in myocardial tissue (blue) and increased cross-sectional cardiomyocyte areas. These changes were significantly ameliorated after AVLE treatment, indicating myocardial fibrosis suppression and reduced cardiomyocyte hypertrophy (Figure 2E). These changes suggest that AVLE improves myocardial remodeling in db/db mice.

In the MOD group, SOD and GSH levels in cardiac tissue were significantly reduced, while MDA levels and inflammatory cytokine levels were markedly elevated. Following AVLE intervention, SOD and GSH contents in cardiac tissue were significantly elevated, while MDA contents were reduced, TNF-α and IL-6 levels were reduced (*p* < 0.05, Figure 2F–I,K), indicating that AVLE alleviates oxidative stress and inflammatory responses in db/db mice.

### 3.3. Proteomics Analysis of AVLE Treatment for Diabetic Cardiomyopathy

**Figure 3:**
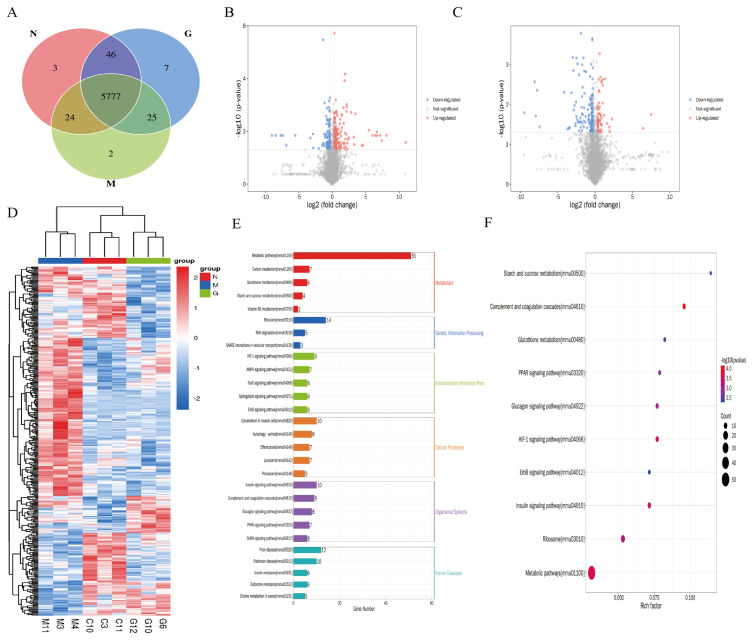
Proteomics analysis of AVLE against DCM. (**A**) Venn diagram; (**B**) Volcano plot showing the upregulated and downregulated proteins of CON vs. MOD; (**C**) Volcano plot showing the upregulated and downregulated proteins of MOD vs. AVLE; (**D**) Hierarchical clustering heatmap (N: CON, M: MOD, G: AVLE); (**E**,**F**) KEGG enrichment analysis of differentially expressed proteins.

Using proteomics to analyze differentially expressed proteins, we identified 5884 quantified proteins (Figure 3A). Differential proteins between the CON, MOD, and AVLE groups totaled 364, with the CON and the MOD groups showing 305 differential proteins (193 upregulated, and 112 downregulated), and the MOD and the AVLE groups showing 219 differential proteins (66 upregulated, and 153 downregulated) (Figure 3B,C). Hierarchical clustering analysis of differentially expressed proteins was visualized as a heatmap (Figure 3D). The three groups showed distinct protein expression profiles with high intragroup reproducibility. The MOD group exhibited extensive expression modes opposite to those of the CON group, indicating significant proteomic alterations in DCM. Notably, AVLE intervention shifted these expression patterns toward those of the CON group, suggesting that AVLE partially reverses DCM-induced protein expression abnormalities.

GO and KEGG enrichment analyses revealed that AVLE treatment of DCM was associated with metabolic pathways, glutathione metabolism, insulin signaling, insulin resistance, endocrine resistance, the AMPK pathway, and the HIF-1 pathway (Figure 3E,F). Notably, glutathione metabolism, a key pathway tightly linked to ferroptosis, suggests that AVLE may inhibit ferroptosis by modulating glutathione metabolism to maintain intracellular glutathione homeostasis and enhance the activity of ferroptosis-suppressing enzymes such as GPX4.

### 3.4. Effects of AVLE on Ferroptosis in db/db Mice

**Figure 4:**
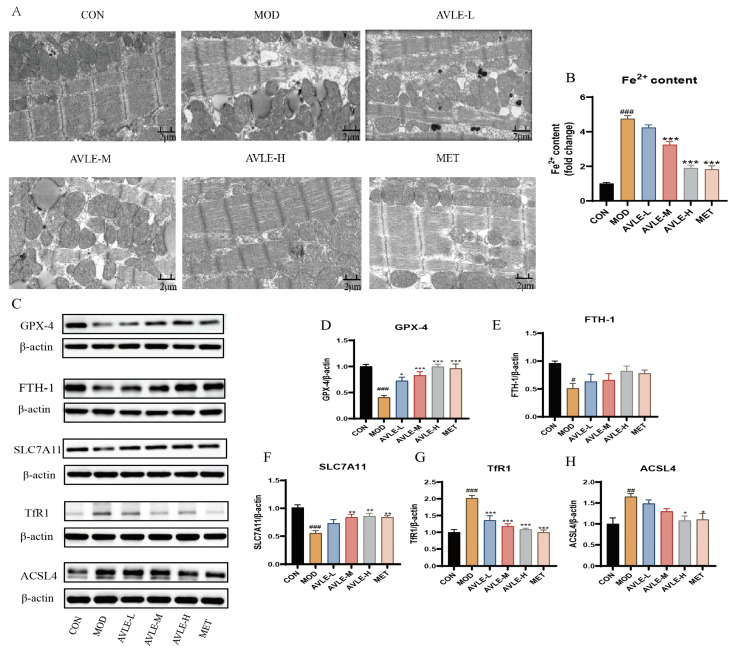
AVLE inhibits mitochondrial injury and ferroptosis in db/db mice. (**A**) Myocardial ultrastructure (Scale bar = 2 μm); Contents of (**B**) Fe^2+^ in myocardial tissues (n = 4); (**C**) Representative blot images and quantification of GPX4 (**D**), FTH1 (**E**), SLC7A11 (**F**), TfR1 (**G**) and ACSL4 (**H**) protein expression in myocardial tissues. Data are reported as mean ± SEM (n = 6, # *p* < 0.05, ## *p* < 0.01, ### *p* < 0.001 vs. CON; * *p* < 0.05, ** *p* < 0.01, *** *p* < 0.001 vs. MOD).

TEM results (×8000) showed (Figure 4A) that, in the CON group, mitochondria exhibited regular morphology with clear cristae and neatly arranged myofilaments. By contrast, in the MOD group, myofilaments were fragmented and disordered, mitochondria were morphologically distorted with ruptured or even vanished cristae, and outer membranes were ruptured; matrix cavities were observed, and numerous lipid droplets were present. Following AVLE treatment, mitochondrial structural disruption was effectively improved, mitochondrial cristae increased, and myofilaments were arranged in order.

Fe^2+^ levels in heart tissue were increased in the MOD group. Following AVLE treatment, Fe^2+^ levels in heart tissue showed a dose-dependent reduction, with significant decreases observed in the AVLE-M, AVLE-H, and MET groups (*p* < 0.05, Figure 4B). This finding indicates that AVLE significantly alleviates mitochondrial structural damage and Fe^2+^ deposition.

WB analysis revealed that the MOD group exhibited markedly reduced protein levels of GPX4, FTH1, and SLC7A11, along with pronounced elevations in TfR1 and ACSL4 expression. AVLE dose-dependently upregulated GPX4, FTH1, and SLC7A11 expression while suppressing TfR1 and ACSL4 levels. Within the AVLE-M, AVLE-H, and MET groups, GPX4 and SLC7A11 expression was markedly elevated (*p* < 0.05, Figure 4D,F), while TfR1 and ACSL4 expression in the AVLE-H and MET groups was significantly decreased (*p* < 0.05, Figure 4G,H). These results indicate that AVLE protects against diabetic cardiomyopathy through suppressing ferroptosis.

### 3.5. Effects of AVLE on HGHF-Induced Cardiomyocyte Injury, Inflammatory Response, and Oxidative Stress in H9c2 Cells

**Figure 5:**
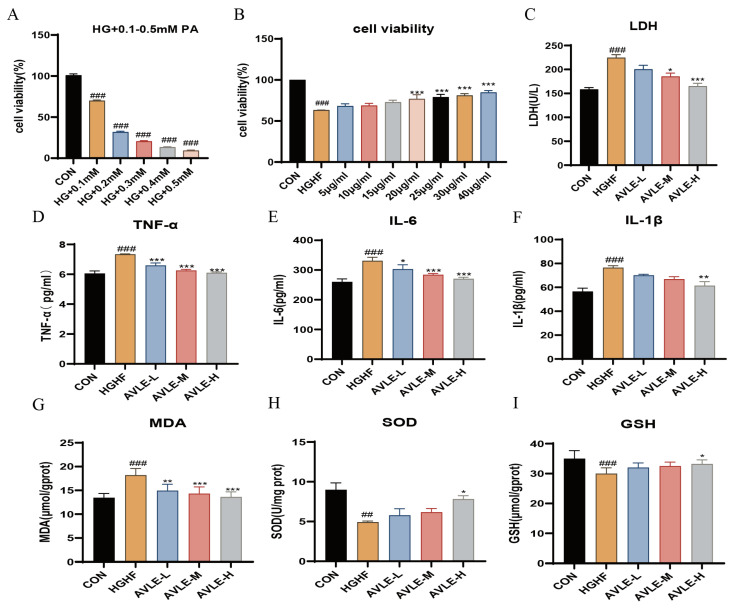
AVLE alleviates HGHF-induced cardiomyocyte injury in H9c2 cells. (**A**) cell viability of H9c2 cells were exposure to high glucose + PA (0.1–0.5 mM) and (**B**) were administered with HGHF + AVLE (0–40 μg/mL); (**C**) LDH levels; Contents of (**D**) TNF-α, (**E**) IL-6, (**F**) IL-1β, (**G**) MDA, (**H**) SOD, (**I**) GSH in H9c2 cells. Data are reported as mean ± SEM (n = 3, ## *p* < 0.01, ### *p* < 0.001 vs. CON; * *p* < 0.05, ** *p* < 0.01, *** *p* < 0.001 vs. HGHF).

To examine the impact of high glucose combined with varying concentrations of palmitic acid (PA) on H9c2 cells, in this experiment used 35 mM glucose supplemented with 0.1 mM–0.5 mM PA for 24 h treatment, followed by cell viability assessment. Results showed that 35 mM glucose + 0.1 mM palmitic acid for 24 h reduced cell viability to 70% (*p* < 0.05, Figure 5A), establishing this as the optimal modeling condition. To assess the impact of AVLE on HGHF-induced injury in H9c2 cells, the cells were incubated with AVLE at increasing doses. The HGHF group exhibited significantly decreased cell viability, following AVLE intervention, cell viability exhibited a gradual elevation along with increasing dosage, with significant increases found in the range of 20–40 μg/mL (*p* < 0.05, Figure 5B). Subsequent experiments thus defined 10 μg/mL as the low dose (AVLE-L), 20 μg/mL as the medium dose (AVLE-M), and 40 μg/mL as the high dose (AVLE-H). Following treatment with AVLE, LDH release rates exhibited a dose-dependent decrease across all groups. Notably, LDH release rates in the AVLE-M and AVLE-H groups were markedly reduced relative to the HGHF group (*p* < 0.05, Figure 5C). These results suggest that AVLE alleviates HGHF-induced injury in H9c2 cells.

To examine the impact of AVLE on inflammation levels in H9c2 cells induced by HGHF, the TNF-α, IL-6 and IL-1β levels were determined. HGHF exposure markedly elevated the production of these cytokines. Following AVLE intervention, TNF-α and IL-6 levels were markedly decreased in all treatment groups, while IL-1β levels reduced markedly in the AVLE-H group (*p* < 0.01, Figure 5D–F). This finding suggests that AVLE attenuates inflammation levels in HGHF-induced H9c2 cells.

The impact of AVLE on oxidative stress in HGHF-induced H9c2 cells was evaluated by measuring intracellular MDA, SOD, and GSH levels. As shown in Figure 5, MDA levels were markedly elevated by HGHF exposure, whereas SOD and GSH contents were reduced. AVLE dose-dependently decreased MDA content (*p* < 0.05, Figure 5G); SOD and GSH contents were notably elevated in the AVLE-H group (Figure 5H,I), suggesting that AVLE alleviates oxidative stress in HGHF-induced H9c2 cells.

### 3.6. Effects of AVLE on Ferroptosis in HGHF-Induced H9c2 Cells

**Figure 6:**
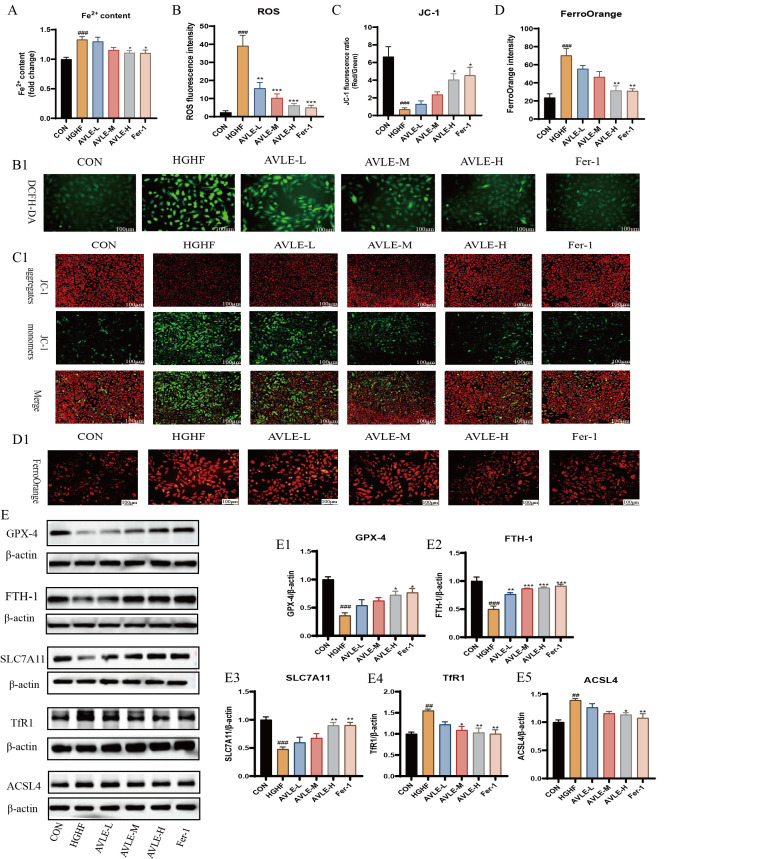
AVLE inhibits ferroptosis in HGHF-induced H9c2 cells. Contents of (**A**) Fe^2+^ in H9C2 cells. (**B1**) Representative images and (**B**) fluorescence intensity of DCFH-DA. (**C1**) Representative images and (**C**) JC-1 fluorescence ratio reflecting MMP. (**D1**) The images and (**D**) fluorescence intensity of FerroOrange (scale bar = 100 μm); (**E**) representative blot images and the expression levels of GPX4 (**E1**), FTH-1 (**E2**), SLC7A11 (**E3**), TfR1 (**E4**) and ACSL4 (**E5**) in H9c2 cells. Data are reported as mean ± SEM (n = 3, ## *p* < 0.01, ### *p* < 0.001 vs. CON; * *p* < 0.05, ** *p* < 0.01, *** *p* < 0.001 vs. HGHF).

Ferrostatin-1 (Fer-1), a ferroptosis inhibitor that blocks the ferroptosis process by eliminating lipid radicals [22]. To further examine whether the cardioprotective effects of AVLE are mediated through ferroptosis inhibition, Fer-1 was added as a positive control in the experiment. ROS were detected using DCFH-DA probes. HGHF exposure significantly elevated ROS fluorescence intensity, while AVLE and Fer-1 significantly reduced fluorescence intensity (*p* < 0.05, Figure 6(B,B1)). This result indicates that AVLE inhibits ROS production in HGHF-induced H9c2 cells.

Fluorescent staining with JC-1 is a commonly used method for assessing mitochondrial membrane potential (MMP). When MMP is elevated, JC-1 emits red fluorescence, but dissociates into green fluorescent monomers when MMP is low [23]. MMP alterations were detected by calculating the red/green fluorescence intensity ratio. Staining results showed that MMP in the HGHF group was significantly reduced, indicating mitochondrial damage. MMP was significantly increased after AVLE and Fer-1 treatment (*p* < 0.05, Figure 6(C,C1)), suggesting that AVLE exerts cardioprotective effects by ameliorating mitochondrial damage.

Ferroptosis relies on the catalytic action of iron ions. Fe^2+^ levels in cells were assessed with a ferrous reagent kit and fluorescent probe to observe iron deposition in H9c2 cells. FerroOrange is a fluorescent probe specifically designed to detect unstable ferrous ions (Fe^2+^), with its fluorescence intensity accurately reflecting iron ion content. Results showed that the HGHF group exhibited significantly elevated average fluorescence intensity and ferrous ion content. AVLE and Fer-1 significantly reduced both Fe^2+^ levels and fluorescence intensity (*p* < 0.05, Figure 6(D,D1)), indicating that AVLE and Fer-1 inhibit HGHF-induced iron deposition in H9c2 cells.

Western blot was performed to assess ferroptosis-related proteins in all experimental groups. The data demonstrated that HGHF exposure led to a marked reduction in GPX4, FTH1, and SLC7A11 expression, whereas TfR1 and ACSL4 were markedly upregulated. Treatment with AVLE and Fer-1 effectively reversed these alterations by upregulated GPX4, FTH1, and SLC7A11 and downregulated TfR1 and ACSL4. Specifically, GPX4, FTH1, and SLC7A11 expression in the AVLE-M, AVLE-H, and Fer-1 groups was upregulated (*p* < 0.05, Figure 6(E1–E3)), TfR1 and ACSL4 expression in the AVLE-H and Fer-1 groups was significantly downregulated (Figure 6(E4,E5)). This suggests that AVLE mitigates HGHF-induced H9c2 cell injury by suppressing ferroptosis.

## 4. Discussion

Diabetic cardiomyopathy (DCM) is a microvascular complication of diabetes, increasingly recognized as a serious condition that contributes substantially to the burden of disability and mortality in diabetic patients. Hyperglycemia exerts myocardial toxicity through both direct and indirect mechanisms during DCM development, triggering cardiac remodeling and impairing diastolic and systolic function, ultimately leading to severe heart failure. The mechanisms underlying DCM are complex and multifaceted: disruption of normal glucose and lipid metabolism ultimately resulting in insulin resistance, and this metabolic disturbance exacerbates several cellular dysfunctions, including mitochondrial dysfunction and stress on the endoplasmic reticulum. In addition, inflammatory responses are activated and calcium homeostasis is dysregulated, all of which further compromise heart function [5,24,25]. Because of its intricate mechanisms involving multiple interacting factors, significant unmet needs persist in developing therapeutic strategies targeting its key pathological pathways. Growing evidence confirms that ferroptosis contributes to DCM development. Ferroptosis, driven by lipid peroxidation, is an iron-dependent form of programmed cell death differing from apoptosis and necrosis in its molecular mechanisms [26]; it is associated with iron and lipid metabolism, initiated by a range of physiological factors and pathological stresses [27].

AVLE exhibits significant pharmacological activities, particularly due to its flavonoid content, including anti-inflammatory [28], antioxidant [29], antihypertensive [30], hypolipidemic [21], and cardioprotective effects [18,31]. Research by Si et al. [21] indicates that AVLE improves lipid metabolism, alleviates oxidative stress, and reduces hepatic lipid accumulation through the activation of the NRF2 and AMPK pathways. Zhang et al. [31] found that AVLE provides cardioprotection by modulating the AKT/Bcl-2 pathway to inhibit DOX-induced cardiotoxicity, oxidative stress, and apoptosis. Yuan et al. [32] demonstrated AVLE’s antihyperglycemic, antihyperlipidemic, and antioxidant effects in type 2 diabetic mice. To determine whether AVLE prevents DCM by suppressing ferroptosis, we established in vivo model using db/db mice and in vitro by exposing H9c2 cardiomyocyte to HGHF, to evaluate its protective effects and explore underlying mechanisms.

Metabolic environment changes linked with diabetes, including lipotoxicity, glucotoxicity, and altered insulin signaling, have become key pathogenic contributors in DCM [33]. In the in vivo experiment, MOD group mice had significantly higher blood glucose and body weight than CON group mice, along with markedly increased serum TG, TC, and LDL-C levels. Although AVLE failed to ameliorate blood glucose and body weight, it decreased serum TG, TC, and LDL-C levels, alleviating lipid accumulation—consistent with findings by Si et al. [21]. CK-MB, LDH, and cTnT serve as primary biochemical markers for myocardial injury assessment [34], as their levels markedly increase under severe injury conditions [35,36]. Furthermore, during DCM development, systolic dysfunction is commonly accompanied by diastolic dysfunction [33], AVLE reduced serum levels of myocardial injury markers, improved heart systolic–diastolic function, and reversed myocardial remodeling. Histopathological analysis further revealed that AVLE markedly alleviated myocardial cell disarray, interstitial collagen deposition, and cardiomyocyte hypertrophy. These findings confirm that AVLE may exert cardioprotective effects by improving cardiac functional impairment through mechanisms independent of glucose reduction.

Considerable evidence supports that oxidative stress, inflammatory, and fibrosis are pivotal mechanisms in DCM development [37,38,39]. Malondialdehyde (MDA) is the terminal product of lipid peroxidation, whereas GSH and SOD exert antioxidant effects by removing free radicals and reducing MDA [40]. In db/db mice, we observed significantly reduced SOD activity and GSH content in cardiac tissues of MOD mice, while MDA and inflammatory markers were markedly increased. AVLE mitigated oxidative stress and inflammatory responses in cardiac tissues of db/db mice. Based on the in vivo studies, we aimed to further explored the potential mechanisms of AVLE in preventing diabetic cardiomyopathy by cultivating H9c2 cells in HGHF condition to model diabetic myocardial injury. In vitro results demonstrated that AVLE provided significant protection against HGHF-induced cellular damage, as evidenced by enhanced cell viability, preserved cell membrane integrity, and alleviated cellular oxidative stress and inflammation levels. AVLE protects against cardiac injury by reducing inflammation and attenuating oxidative stress both in vivo and in vitro.

Emerging research supports that ferroptosis participates in the progression of DCM [13,15,41]. This iron-dependent cell death process is marked by extensive lipid peroxidation. Unlike typical forms of unprogrammed cell death, this process is primarily associated with disruptions in iron metabolism, leading to an increase in lipid peroxidation. Additionally, it is associated with an imbalance within the antioxidant systems of the cell [42]. Iron, as a vital trace element, participates in numerous physiological processes. To maintain adequate levels of iron for physiological functions while minimizing its toxic effects, iron homeostasis is tightly regulated [43]. By inducing ROS production and modulating lipid peroxidation, iron overload can trigger ferroptosis. When cellular Fe^2+^ levels are elevated, the Fenton reaction involving free Fe^2+^ and lipid peroxides generates ROS [44]. FTH1, as an iron storage protein, its upregulation can limit intracellular free Fe^2+^ levels, thereby reducing ROS and lipid peroxidation mediated by Fenton reaction [45]. Mitochondria, as the primary organelles for energy metabolism and ROS production, serve a critical function in cardiomyocytes [46]. As key players in iron metabolism and ferroptosis, mitochondria exhibit reduced volume and membrane density, disrupted cristae, and ruptured outer membranes during ferroptosis [47]. These morphological changes are indeed observed in db/db mice. Notably, AVLE reverses mitochondrial structural disruption and reduces Fe^2+^ levels in db/db mice. In vitro, AVLE also decreases Fe^2+^ and ROS levels in H9c2 cardiomyocytes while enhancing mitochondrial membrane potential (MMP), and upregulates FTH1 expression both in vivo and in vitro. The regulation of AVLE on these indicators exhibits a consistent trend with those of the ferroptosis inhibitor Fer-1, suggesting that its mechanism of action is intimately correlated with the inhibition of ferroptosis. These findings indicate improvement in iron metabolism disorders and mitochondrial damage, thereby inhibiting ferroptosis and exerting cardioprotective effects in DCM.

The Xc^−^/GSH/GPX4 axis represents the primary pathway for ferroptosis development, as the core antioxidant system regulating intracellular ferroptosis, it protects cells from ferroptosis damage by maintaining the cascade reaction of cystine uptake, GSH synthesis, and lipid peroxide clearance [48]. The Xc^−^ system functions as a cysteine/glutamate reverse transport system composed of two distinct proteins—solute carrier family 7 member 11 (SLC7A11) and 3 member 2 (SLC3A2)—embedded in the phospholipid bilayer [49]. In this system, cystine uptake mediated by system Xc^−^ represents the key regulatory step for GSH synthesis; after being transported into cells, cystine is converted into cysteine and further participates in the synthesis of GSH [50]. Previous studies have confirmed that inhibiting system Xc^−^ transporter activity triggers ferroptosis by depleting GSH. GSH functions not only as a critical cofactor for GPX4 enzymatic activity but also acts as the primary non-enzymatic antioxidant in cells, maintaining intracellular redox homeostasis through its sulfhydryl groups. On this basis, GPX4 utilizes the reducing equivalents provided by GSH to convert toxic lipid hydroperoxides into non-toxic lipid alcohols, attenuating the accumulation of lipid peroxides [51]. Our results indicate that during DCM, GSH levels significantly decrease, while GPX4 and SLC7A11 expression is markedly downregulated, suggesting suppression of the Xc^−^/GSH/GPX4 axis. Conversely, expression of the transferrin receptor TfR1 and a critical marker of lipid peroxidation accumulation, ACSL4, was upregulated. Notably, ACSL4 provides reaction substrates for lipid peroxide generation by catalyzing the esterification of polyunsaturated fatty acids [52], whereas GPX4 is responsible for clearing generated lipid peroxides, the two forming a “substrate supply-product clearance” synergistic regulatory couple [53].

KEGG pathway enrichment analysis further revealed the molecular network underlying the regulation of ferroptosis by AVLE. The marked enrichment of the glutathione metabolism pathway closely aligns with the activation of the Xc^−^/GSH/GPX4 axis, confirming the core status of the Xc^−^/GSH/GPX4 axis at the systems level. This pathway encompasses key processes including cystine uptake, GSH biosynthesis, and GPX4-mediated lipid peroxide clearance [54]. Therefore, the enrichment of the glutathione metabolism pathway not only corroborates our experimental findings that AVLE upregulates SLC7A11 and GPX4 expression and restores GSH levels, but also provides system-level evidence from the holistic metabolic network perspective that AVLE inhibits ferroptosis by modulating the Xc^−^/GSH/GPX4 axis. Additionally, the enrichment of the PPAR signaling pathway provides a potential upstream regulatory explanation for the downregulation of ACSL4. PPARγ, as a core member of this pathway, is a key transcription factor regulating lipid metabolism, inflammation, and oxidative stress [55]. Recent research has revealed that PPARγ activation can inhibit ferroptosis through various pathways, including regulating ACSL4 expression and suppressing lipid peroxidation [56]. Tang et al. [41] demonstrated that irisin suppresses iron accumulation and lipid peroxidation by inhibiting p53, thereby upregulating GPX4 and SLC7A11 expression and mitigating myocardial injury in an STZ-induced type 1 diabetes model. Our findings similarly demonstrate that AVLE significantly upregulates GPX4, SLC7A11, and FTH-1 expression while downregulating TfR1 and ACSL4 expression in the T2DM model, comparable to the effect of Fer-1 in suppressing ferroptosis. These results further support that AVLE exerts cardioprotective effects in diabetes by inhibiting ferroptosis through modulating the Xc^−^/GSH/GPX4 axis.

## 5. Conclusions

In summary, both in vivo and in vitro studies have demonstrated the protective effects of AVLE against diabetic cardiomyopathy, highlighting its roles in regulating dyslipidemia, improving cardiac function, and exhibiting antioxidant and anti-inflammatory properties. This study elucidates how AVLE exerts cardioprotective effects against diabetic myocardial injury by inhibiting ferroptosis through multiple pathways. Results indicate that AVLE exerts cardioprotective effects against diabetic cardiomyopathy by reducing oxidative stress and inflammation levels in db/db mice and HGHF-induced H9c2 cells, improving lipid peroxidation and mitochondrial damage, and regulating the Xc^−^/GSH/GPX4 axis to inhibit ferroptosis. Our findings not only demonstrate the therapeutic potential of AVLE against DCM but also provide a conceptual advance for exploring natural products in the management of diabetic complications.

## Data Availability

The original contributions presented in this study are included in the article/Supplementary Materials. Further inquiries can be directed to the corresponding author.

## Supplementary Materials

The following supporting information can be downloaded at: https://www.mdpi.com/article/10.3390/cimb48040375/s1.

## Abbreviations

The following abbreviations are used in this manuscript:

T2DM	Type 2 diabetes mellitus
DCM	Diabetic cardiomyopathy
AVLE	*Apocynum venetum* L. leaves extract
MET	Metformin
PA	Palmitic acid
HGHF	High-glucose and high-fat
Fer-1	Ferrostatin-1
FBG	Fasting blood glucose
LDH	Lactate dehydrogenase
CK-MB	Creatine Kinase-Myocardial Band
cTnT	Cardiac troponin T
LVIDs	Left ventricular internal dimension at end-systole
LVIDd	Left ventricular internal dimension at end-diastole
LVEF	Left ventricular ejection fraction
LVFS	Left ventricular fractional shortening
TC	Total cholesterol
TG	Triglyceride(s)
LDL-C	Low-density lipoprotein cholesterol
HDL-C	High-density lipoprotein cholesterol
MDA	Malondialdehyde
SOD	Superoxide dismutase
GSH	Glutathione
ROS	Reactive oxygen species
IL-6	Interleukin-6
IL-1β	Interleukin-1 beta
TNF-α	Tumor necrosis factor-alpha
TEM	Transmission electron microscopy
GPX-4	Glutathione peroxidase 4
FTH-1	Ferritin heavy chain 1
SLC7A11	Solute carrier family 7 member 11
TfR1	Transferrin receptor 1
ACSL4	Acyl-CoA synthetase long-chain family member 4
MMP	Mitochondrial membrane potential
DMEM	Dulbecco’s modified Eagle’s medium
FBS	Fetal Bovine Serum
H&E	Hematoxylin and eosin
WGA	Wheat germ agglutinin
PBS	Phosphate-buffered saline
TBST	Tris-buffered saline with Tween
