# Supplementary material for: Protective Effect of Apocynum venetum L. Leaves Extract Against Diabetic Cardiomyopathy: Inhibition of Oxidative Stress and Ferroptosis via Modulation of the Xc−/GSH/GPX4 Axis
Source: Curr Issues Mol Biol. 2026 Apr 3;48(4):375. doi: 10.3390/cimb48040375 (PMC13115175; doi:10.3390/cimb48040375)
Supplement: Supplementary file 1 [file cimb-48-00375-s001.zip › cimb-4182658-supplementary.pdf]

Supplementary Materials: Chemical Characterization of *Apocynum venetum* L. Leaves Extract (AVLE) by UHPLC-Q Exactive Orbitrap-HRMS Analysis

The chromatographic profiles and tentative compound annotations summarized here were derived from UHPLC-Q Exactive Orbitrap-HRMS data previously reported by our research group (Si et al., 2025, *Food Research International*, https://doi.org/10.1016/j.foodres.2025.117326) and are provided to support the chemical characterization of AVLE.

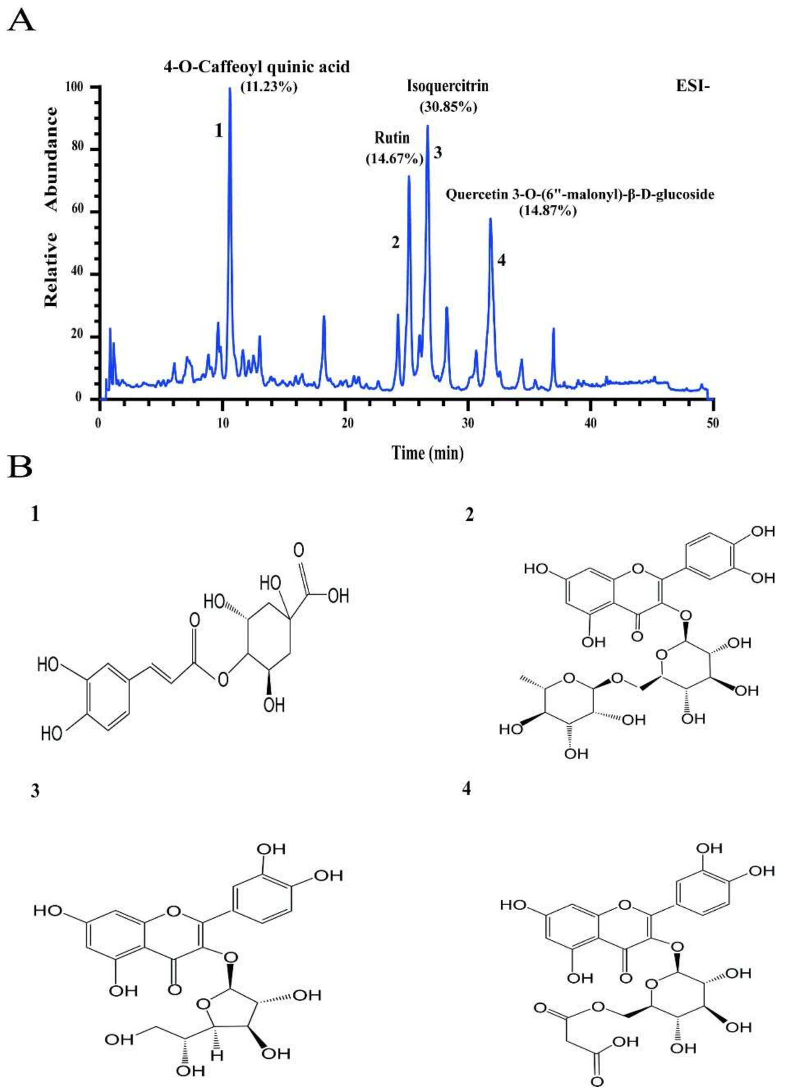

Figure S1. Identification of chemical constituents in AVLE by UHPLC-Q Exactive Orbitrap-HRMS analysis. (A) UHPLC-Q Exactive Orbitrap-HRMS chromatogram of AVLE in negative ionization mode. (B) The chemical structures of the main components in AVLE: 4-O-caffeoyl quinic acid, rutin, isoquercitrin, and quercetin-3-O-(6"-O-malonyl)-β-D-glucoside.

To address the editorial request for chemical characterization, the tentative composition of AVLE was summarized using previously acquired UHPLC-Q Exactive Orbitrap-HRMS data. Analysis was performed in negative electrospray ionization mode, enabling the detection of a wide range of polar and semi-polar metabolites. The representative base peak intensity (BPI) chromatogram (Figure S1A) revealed complex yet reproducible metabolic profiles, reflecting the heterogeneous nature of AVLE.

Compound identification was conducted based on accurate mass measurements and MS/MS fragmentation patterns, with annotations supported by spectral library matching and comparison with reference substances. Using this approach, a total of 62 compounds were identified in AVLE, including 41 flavonoids, 17 phenolic acids, 3 coumarins, and 1 caffeic acid ester (Table S1). The quantitative analysis revealed that the contents of 4-O-caffeoyl quinic acid, rutin, isoquercitrin, and quercetin-3-O-(6"-O-malonyl)-β-D-glucoside in AVLE were 11.23%, 14.67%, 30.85%, and 14.87%, respectively. The chemical structures of these main components are presented in Figure S1B. These compounds are noted for their antioxidant properties and have been suggested to play a role in the prevention of metabolic disorders.

Table S1. Identifications of compounds of AVLE with UHPLC-Q Exactive Orbitrap-HRMS.

No	t_R (min)	$[M-H]^-$ (m/z)	Error (ppm)	Formula	MS²data(m/z)	Identification
1	1.23	191.05604	4.43	$C_6H_8O_7$	173(10),111(10),85(30)	Quininic acid
2	1.27	341.10867	2.45	$C_{12}H_{22}O_{11}$	179(50),119(60),89(100)	Caffeoylglucose
3	2.80	169.01421	4.39	$C_7H_6O_5$	125(100)	Gallic acid
4	5.42	305.06689	4.31	$C_{15}H_{14}O_7$	261(5),203(5),167(30),125(100)	Gallocatechin
5	5.97	339.07266	4.73	$C_{15}H_{16}O_9$	177(100),149(5),133(10),105(5)	Esculin hydrate
6	6.05	177.01921	4.37	$C_9H_6O_4$	147(5),133(40),105(40)	Aesculetin
7	6.08	353.08789	-2.01	$C_{16}H_{18}O_9$	191(100),179(70),135(60)	Chlorogenic acid
8	6.39	339.07233	3.74	C15H16O9	177(100),161(10),133(20),105(5)	Esculin hydrate isomer
9	8.33	593.12872	-0.41	$C_{30}H_{26}O_{13}$	467(10),425(40),305(5),289(60),125(100)	Gallocatechin-(4,8)-catechin
10	8.47	337.09350	4.35	$C_{16}H_{18}O_8$	191(20),173(5),163(100),135(5),119(50)	Coumaryl quinic acid
11	8.91	401.14505	2.06	$C_{18}H_{26}O_{10}$	269(100),161(70),143(100)	Apigenin-arabinoside
12	9.76	179.03517	4.54	$C_9H_8O_4$	135(100)	Caffeic acid
13	8.39	305.06699	4.61	$C_{15}H_{14}O_7$	261(10),203(5),167(30),125(100)	Gallocatechin isomer
14	9.85	353.08620	-1.41	$C_{16}H_{18}O_9$	191(50),179(60),173(100),135(60)	5-O-caffeoylquinic acid
15	10.44	191.05559	4.97	$C_7H_{11}O_6$	173(5),85(30)	Quininic acid isomer
16	10.63	353.08690	0.58	$C_{16}H_{18}O_9$	191(100)	4-0-caffeoyl quinic acid
17	10.93	625.14185	-2.54	$C_{27}H_{30}O_{17}$	463(40),299(100),271(70),243(5),151(5)	Quercetin-3-0-sophoroside isomer
18	11.68	625.14154	2.58	$C_{27}H_{30}O_{17}$	463(50),299(100),271(70),243(5),151(5)	Quercetin-3-0-sophoroside
19	12.09	771.19995	2.74	$C_{33}H_{40}O_{21}$	609(50),299(100),271(80),255(5),243(10),151(20)	Quercetin-O-rutinyl-glucoside
20	12.30	289.07208	4.91	$C_{15}H_{14}O_6$	245(30),203(50),123(90),109(100)	Catechin
21	12.96	337.09305	3.72	$C_{16}H_{18}O_8$	191(5),173(100),163(300)	Coumaryl quinic acid isomer
22	13.91	667.15253	3.05	$C_{29}H_{32}O_{18}$	505(30),463(30),299(100),271(70),243(5),151(5)	Quercetin-O-acetyl-glucose-glucoside
23	14.07	367.10400	4.48	$C_{17}H_{20}O_9$	193(10),173(100),135(20)	Feruloylquinic acid
24	14.28	337.09300	3.63	$C_{16}H_{18}O_8$	191(100),173(10),163(20),145(5)	Coumaryl quinic acid isomer
25	14.67	667.15240	2.86	$C_{29}H_{32}O_{18}$	607(5),547(5),505(30),463(30),299(100),271(70),243(5),151(5)	Quercetin-acetyl-glucose-glucoside
26	14.74	337.09271	2.72	$C_{16}H_{18}O_8$	191(100),173(10),163(5),145(5)	Coumaryl quinic acid isomer
27	14.92	337.09320	4.26	$C_{16}H_{18}O_8$	191(100),173(10),163(10),145(5)	Coumaryl quinic acid isomer
28	15.48	367.10358	4.48	$C_{17}H_{20}O_9$	193(100),173(10),135(20)	Feruloylquinic acid isomer
29	16.59	625.14160	2.68	$C_{27}H_{30}O_{17}$	316(50),299(40),271(40),243(5),165(5)	Myricetin-O-rutinoside
30	19.87	625.14209	-2.15	$C_{27}H_{30}O_{17}$	316(80),300(10),287(30),271(40),243(5),151(5)	Myricetin-O-rutinoside
31	20.08	479.08368	3.47	$C_{21}H_{20}O_{13}$	316(100),287(30),271(40),179(5),151(10)	Myricetin-O-glucoside
32	20.71	479.08340	2.89	$C_{21}H_{20}O_{13}$	316(100),287(40),271(50),179(10),151(5)	Myricetin-O-glucoside isomer
33	22.90	463.08914	4.41	$C_{21}H_{20}O_{12}$	301(100),255(5),227(5),179(5),151(50)	Quercetin-O-hexosideisomer
34	24.07	271.06110	3.68	$C_{15}H_{12}O_5$	243(5),151(90),119(80),107(30)	Trihydroxyflavone
35	24.31	609.14620	1.92	$C_{27}H_{30}O_{16}$	300(100),271(60),255(30),227(10),179(10),151(10)	Quercetin-3-0-galactosyl-(1→6)-0-rhamnoside
36	25.20	609.14620	1.92	$C_{27}H_{30}O_{16}$	300(100),271(60),255(30),243(10),227(10),179(5),151(15)	Rutin
37	25.38	463.08881	3.68	$C_{21}H_{20}O_{12}$	300(100),271(90),255(40),227(5),179(10),151(20)	Quercetin-O-hexoside
38	26.09	463.08853	3.09	$C_{21}H_{20}O_{12}$	300(100),271(90),255(30),243(30),179(10),151(20)	Hyperin
39	26.73	463.08841	2.82	$C_{21}H_{20}O_{12}$	300(90),271(100),255(40),243(20),179(10),151(20)	Isoquercitrin
40	27.43	593.15149	2.35	C2₇H₃₀O₁₅	447(5),284(60),255(50),227(30),151(5)	Kaempferol-O-glucose-rhamnoside
41	27.50	463.08926	4.67	$C_{21}H_{20}O_{12}$	300(100),271(70),255(40),227(10),179(10),151(20)	Quercetin-O-hexoside isomer
42	28.29	477.06705	1.42	$C_{21}H_{18}O_{13}$	301(100),273(10),255(20),179(30),151(60)	Quercetin-3-0-glucuronide
43	29.29	515.12048	4.03	$C_{25}H_{24}O_{12}$	353(70),191(60),179(50),173(100),161(40),135(70)	Dicaffeoylquinic acids
44	30.09	447.09387	3.76	$C_{21}H_{20}O_{11}$	284(50),255(90),227(70),151(5)	Trifolin

45	30.68	593.15167	2.66	$C_{27}H_{30}O_{15}$	284(100),255(50),227(30),151(5)	Kaempferol-3-0-rutinoside
46	31.82	447.09406	4.18	$C_{21}H_{20}O_{11}$	284(60),255(70),227(60),151(5)	Astragalin
47	31.88	549.08813	1.16	$C_{24}H_{22}O_{15}$	505(90),300(100),271(70),255(40),243(20),151(30)	Quercetin-3-0-(6"-malonyl)-β-D-glucoside
48	31.45	515.11951	2.14	$C_{25}H_{24}O_{12}$	353(70),191(100),179(50),173(10),135(50)	Dicaffeoylquinic acids isomer
49	32.59	549.08899	2.72	$C_{24}H_{22}O_{15}$	505(80),300(100),271(70),255(30),243(20),151(20)	Quercetin-3-0-(6"-malonyl)-β-D-galactoside pyranoside
50	32.11	447.09790	3.56	$C_{21}H_{20}O_{11}$	284(50),255(100),227(90)	Kaempferol-O-hexoside
51	34.42	505.09924	3.12	$C_{23}H_{22}O_{13}$	300(100),271(90),255(30),243(20),227(10),179(10),151(20)	Quercetin-0-acetyl-glucoside
52	35.37	505.09940	3.42	$C_{23}H_{22}O_{13}$	300(100),271(70),255(40),243(20),227(5),179(10),151(20)	Quercetin-O-acetyl-galactoside
53	35.51	549.08899	2.72	$C_{24}H_{22}O_{15}$	505(90),463(10),300(100),271(60),255(30),243(20),179(10),151(5)	Quercetin-O-malonyl-glucoside
54	35.54	515.11896	3.31	$C_{25}H_{24}O_{12}$	353(90),191(50),179(60),173(100),135(60)	Dicaffeoylquinic acids isomer
55	35.90	533.09360	1.96	$C_{24}H_{22}O_{14}$	489(70),284(100),255(70),227(40)	Kaempferol-O-malonyl-glucoside
56	35.97	489.10455	3.68	$C_{23}H_{22}O_{12}$	447(5),284(100),255(70),227(50),187(5),151(5)	Acetyl-kaemphenol-0-glucoside
57	36.47	505.09961	-3.10	$C_{23}H_{22}O_{13}$	463(5),300(100),271(90),255(40),243(20),227(10),179(10),151(20)	Acetyl-quercetin-O-glucoside
58	36.92	489.10471	3.99	$C_{23}H_{22}O_{12}$	285(100),255(70),227(40),187(5),133(5)	Acetyl-luteolin-O-glucoside
59	36.99	533.09369	2.08	$C_{24}H_{22}O_{14}$	489(50),285(100),255(50),227(20),131(5)	Luteolin-O-malonyl-glucoside
60	37.55	489.10458	3.74	$C_{23}H_{22}O_{12}$	284(100),255(80),227(60),187(5),151(5)	Acetyl-kaemphenol-O-glucoside isomer
61	37.90	489.10461	3.81	$C_{23}H_{22}O_{12}$	284(80),255(100),227(70),185(5),151(5)	Acetyl-kaemphenol-0-glucoside isomer
62	39.46	301.03537	3.62	$C_{15}H_{10}O_{7}$	271(20),229(5),179(40),151(80)	Quercetin

Si L, Chen L, Abdulla R, Aisa HA. Anti-hyperlipidemic effects of *Apocynum venetum* L. leaves extract on high-fat diet-induced hyperlipidemia: Modulation of lipid metabolism and oxidative stress. *Food Research International*. 2025;221:117326. doi:10.1016/j.foodres.2025.117326
